# Association Between Urine pH and Kidney Outcomes in Patients with Biopsy-Proven Kidney Disease

**DOI:** 10.3390/diagnostics16203418

**Published:** 2026-10-20

**Authors:** Kenji Tsuji, Naruhiko Uchida, Hiroyuki Nakanoh, Yoshitaka Inoue, Kazuhiko Fukushima, Shinji Kitamura, Jun Wada

**Affiliations:** Department of Nephrology, Rheumatology, Endocrinology and Metabolism, Graduate School of Medicine, Dentistry and Pharmaceutical Sciences, Okayama University, 2-5-1 Shikata-cho, Okayama 700-8558, Japan

**Keywords:** urine pH, kidney outcomes, medullary casts, global glomerulosclerosis, interstitial fibrosis and tubular atrophy

## Abstract

**Background/Objectives**: Lower urine pH has been associated with reduced kidney function and increased risk of kidney disease, but its prognostic and pathological significance in biopsy-proven kidney disease remains unclear. Medullary cast formation has been shown to predict adverse renal outcomes beyond cortical interstitial fibrosis and tubular atrophy (IFTA), although its clinical determinants are not fully understood. We examined the associations of urine pH with kidney outcomes and medullary cast formation in patients undergoing native kidney biopsy. **Methods**: Among 1167 adults who underwent native kidney biopsy between 2011 and 2024, 503 patients with evaluable medullary tissue were included. Urine pH was analyzed in relation to clinical and histological variables and kidney outcomes. The primary outcome was a ≥40% decline in estimated glomerular filtration rate (eGFR) or initiation of renal replacement therapy. **Results**: Mean baseline eGFR was 60.7 ± 26.2 mL/min/1.73 m^2^ and urine pH was 6.15 ± 0.84. During a median follow-up of 2.11 years, 113 patients (22.5%) reached the outcome. Lower urine pH was associated with higher risk of kidney outcomes. In multivariable Cox models, urine pH remained independently associated with outcomes (hazard ratio per 1-unit increase, 0.69; 95% CI, 0.52–0.92). Addition of urine pH modestly improved discrimination (C-index 0.642 to 0.654; likelihood ratio test, *p* = 0.009). Lower urine pH was associated with greater medullary cast formation in unadjusted analysis. **Conclusions**: Lower urine pH was independently associated with adverse kidney outcomes and with greater medullary cast formation, suggesting that urine pH may reflect intratubular pathological changes associated with CKD progression.

## 1. Introduction

Chronic kidney disease (CKD) is a major global health burden, and identification of prognostic factors remains a critical issue in nephrology [1]. Renal biopsy provides valuable histopathological information that contributes not only to diagnosis but also to prognostic stratification [2]. While cortical lesions have been extensively studied as predictors of renal outcome, the prognostic significance of medullary pathological changes has received comparatively less attention. The renal medulla is particularly vulnerable to hypoxia and tubular obstruction [3,4], suggesting that medullary lesions may have important prognostic implications.

Among tubulointerstitial lesions, tubular cast formation, particularly within the renal medulla, has been proposed as a potential indicator of adverse renal prognosis [5]. Cast formation is thought to reflect intratubular precipitation of proteins, cellular debris, or crystals, potentially contributing to tubular obstruction, increased intratubular pressure, and subsequent tubular injury [5,6,7,8]. In a previous exploratory study from our group, medullary cast formation was associated with adverse kidney outcomes beyond established clinical and histological risk factors [5]. These observations suggest that the medullary microenvironment and tubular factors may contribute to long-term renal outcomes.

Urine pH is a readily available clinical parameter influenced by renal tubular acid–base handling and other factors, including dietary acid load and kidney function [9]. Given its potential influence on protein aggregation and solubility, urine pH may play a role in modulating medullary cast formation. Experimental studies suggest that urinary acid–base conditions may influence protein aggregation and solubility, potentially affecting tubular cast formation [6,10]. However, whether urine pH measured in routine clinical practice reflects these intratubular processes remains uncertain. In addition, acidic tubular conditions have been linked to tubular injury and profibrotic responses [11]. Epidemiological studies suggest that lower urine pH is associated with kidney function decline across diverse populations, including CKD [12,13,14,15,16]. However, the pathological mechanisms underlying this association remain incompletely understood, particularly with respect to medullary cast formation in biopsy-proven kidney disease.

We therefore hypothesized that lower urine pH may be associated with medullary cast formation and adverse kidney outcomes in patients undergoing native kidney biopsy. To test this hypothesis, we conducted a cohort study of patients undergoing native kidney biopsy, examining the association between urine pH, medullary cast formation, and long-term renal outcomes. Elucidating these associations may provide additional clinicopathological insights into kidney disease progression and clarify the potential prognostic value of urine pH in patients undergoing native kidney biopsy.

## 2. Materials and Methods

### 2.1. Study Design and Participants

**Figure 1:**
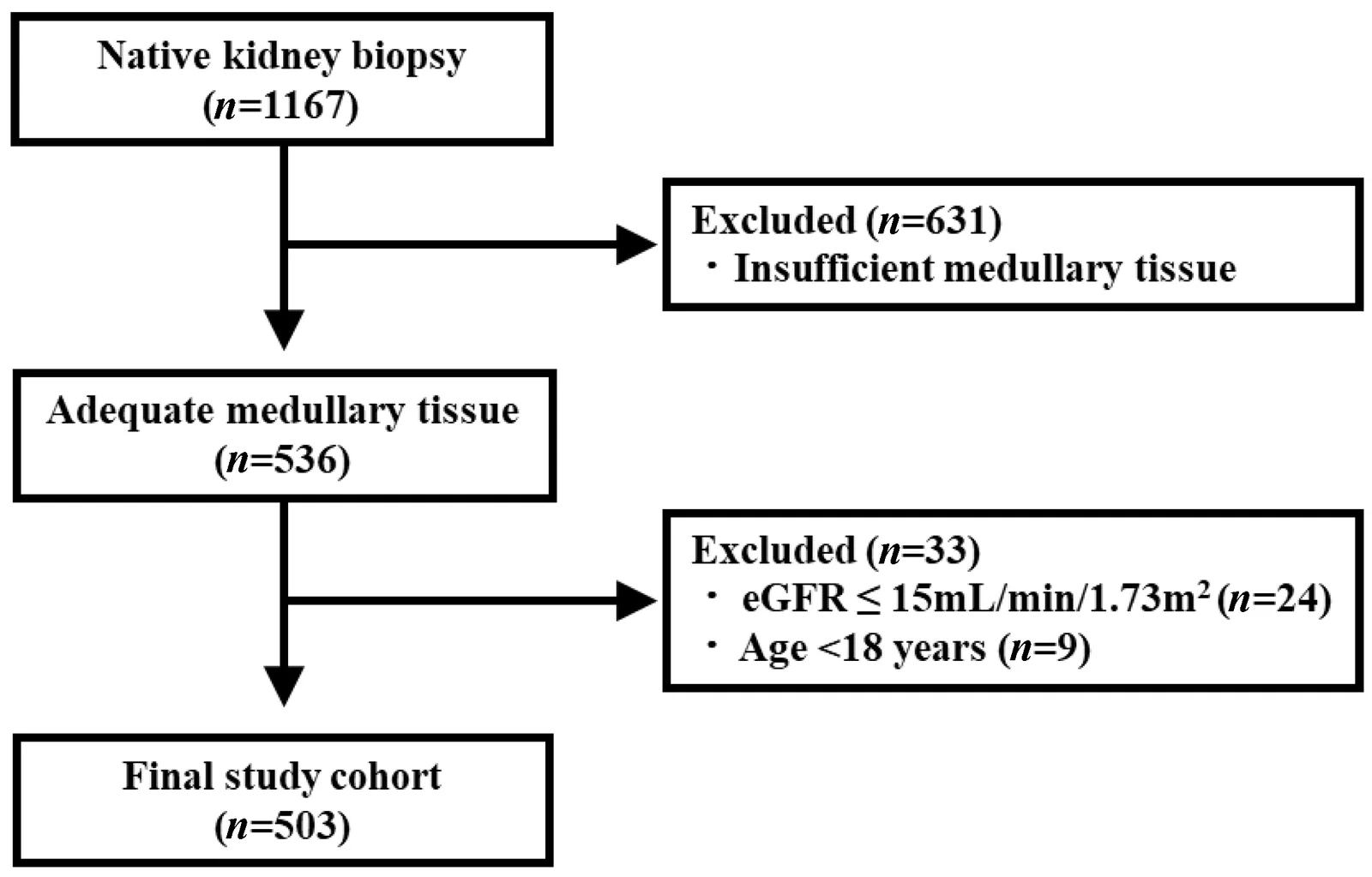
Flowchart of study participants. eGFR, estimated glomerular filtration rate.

We retrospectively reviewed 1167 adult patients who underwent a native kidney biopsy in the Okayama University Hospital (Okayama, Japan) between January 2011 and March 2024. The exclusion criteria were eGFR ≤ 15 mL/min/1.73 m^2^, dialysis at the time of biopsy, age < 18 years, or insufficient medullary tissue. Consequently, 503 patients were enrolled in the study (Figure 1). The study protocol was approved by the ethics committee of Okayama University Hospital Institutional Review Board, Okayama, Japan (approval number: 1908-022 and 2407-038, approved date: 19 July 2019 and 21 June 2024), and is in accordance with the principles of the Declaration of Helsinki. The requirement for written informed consent was waived due to the retrospective nature of the study. Instead, information about the study was publicly disclosed on the institutional website, and patients were provided the opportunity to opt out. The present cohort substantially overlapped with that of our previous study investigating the prognostic significance of medullary cast formation (488 patients) [5]. The present study included an expanded cohort of 503 patients and focused on the association between urine pH and kidney outcomes.

### 2.2. Histological Parameters

**Figure 2:**
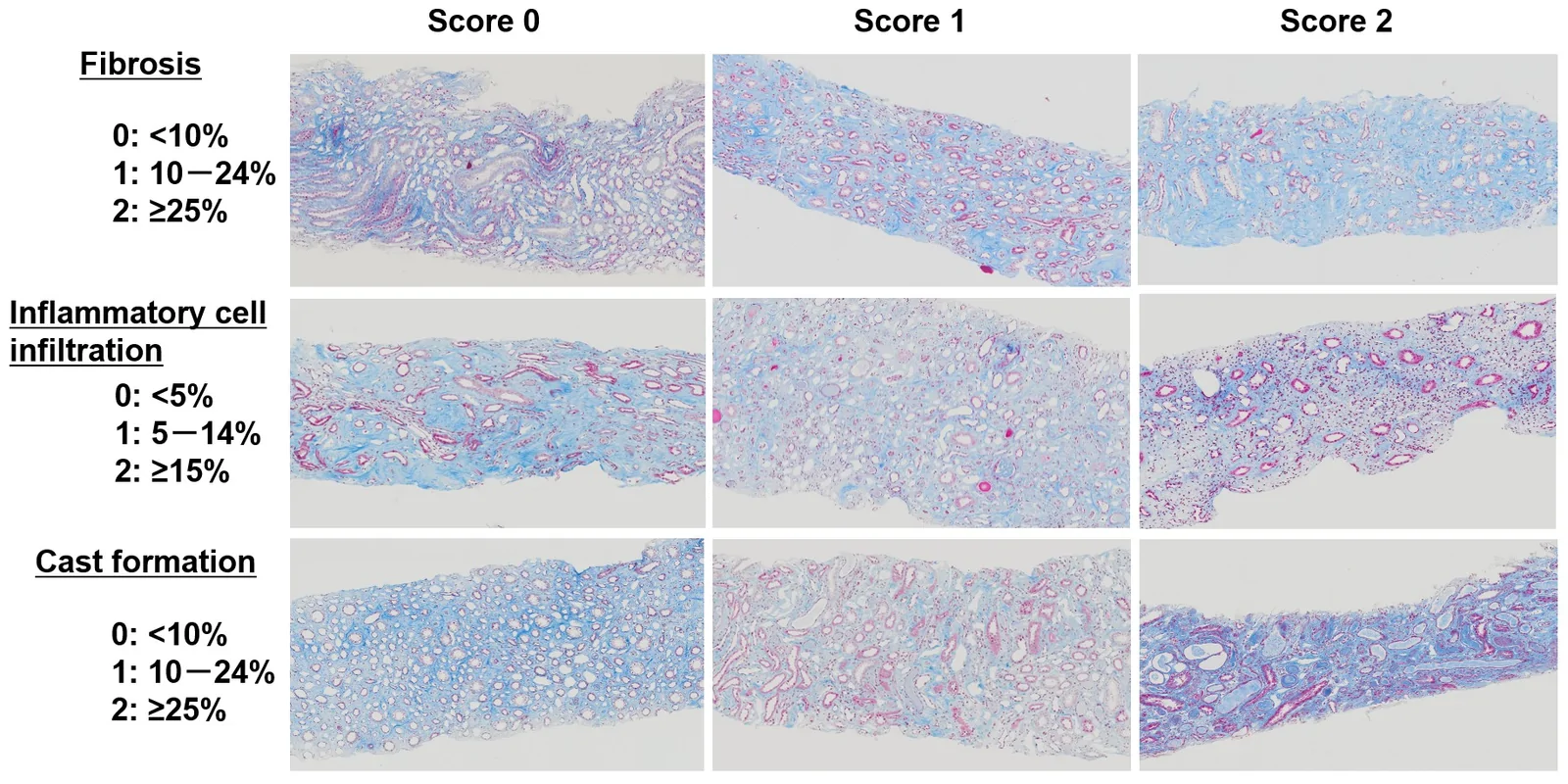
Medullary pathological findings and scoring system. Representative light microscopic images of renal medullary tissue stained with Masson’s trichrome showing medullary fibrosis and cast formation. Each lesion was semi-quantitatively graded as 0, 1, or 2 based on severity. Medullary fibrosis and medullary cast formation were semi-quantitatively graded according to the percentage of affected medullary tissue as follows: score 0, <10%; score 1, 10–24%; and score 2, ≥25%. Representative histological images corresponding to each score are shown. Original magnification: ×40 for all panels.

Obtained kidney tissues were processed for light microscopy, immunofluorescent staining and electron microscopy. For light microscopy, specimens were stained with hematoxylin and eosin, periodic acid-Schiff, periodic acid-methenamine silver and Masson’s trichrome, according to routine methods. All biopsies were adjudicated to determine histologic scores and diagnosis by at least two experienced nephrologists, through conference discussions based on patients’ medical histories, physical information, clinical examinations and pathological findings. The cortical IFTA score were graded on a scale of 0, 1, 2 and 3 (≤10%, 11–25%, 26–50%, >50%, respectively) according to previous reports and the Banff classification of kidney allograft pathology [17]. The percentage of global glomerulosclerosis was calculated as the number of globally sclerotic glomeruli divided by the total number of glomeruli in the biopsy specimen. Global glomerulosclerosis (GS) score was categorized into four grades: 0 (≤10%), 1 (>10% to <25%), 2 (≥25% to <50%), and 3 (≥50%). Adequate medullary tissue was defined as a medullary area of ≥1.5 mm^2^ within the biopsy specimen, and only cases meeting this criterion were included in the medullary analysis. Baseline clinical characteristics were compared between included and excluded patients to assess potential selection bias. Medullary fibrosis and cast formation and inflammation were scored as 0 (<10%), 1 (10–24%), or 2 (≥25%), and inflammatory cell infiltration was scored as 0 (<5%), 1 (5–14%), or 2 (≥15%) (Figure 2), as previously reported [5]. Medullary pathological findings were jointly evaluated by two nephrologists who were blinded to the clinical data. Histopathological scores were determined through consensus discussion.

### 2.3. Clinical Parameters

The following clinicopathologic characteristics were collected at the time of kidney biopsies: age, sex, body mass index (BMI), hemoglobin A1c (HbA1c), the use of angiotensin-converting-enzyme inhibitor (ACE-I) and/or angiotensin II receptor blocker (ARB), blood pressure, history of smoking, serum creatinine (s-Cr) and eGFR levels, urine pH, urinary protein levels and hematuria (>5 erythrocytes per high power field). Urine pH was measured semi-quantitatively using reagent strips and an automated urine analyzer as part of routine laboratory testing at the time of kidney biopsy. Urine samples were processed according to the standard procedures of the hospital’s clinical laboratory. eGFR was evaluated using the equation developed by the Japanese Society of Nephrology [18]. Hypertension was defined as blood pressure (BP) ≥ 140/90 mmHg at the time of the kidney biopsy or use of antihypertensive drugs. HbA1c data are presented as National Glycohemoglobin Standardization Program values according to the recommendations of the Japanese Diabetes Society and the International Federation of Clinical Chemistry [19].

### 2.4. Exposure and Outcomes

The primary exposure was urine pH. Because urine pH was assessed semi-quantitatively using dipstick testing, patients were categorized into clinically relevant urine pH groups (<6.0, 6.0–<7.0, and ≥7.0). Established prognostic factors, including cortical IFTA scores, were included as covariates [2]. The primary endpoint was defined as the first occurrence of either an eGFR decline of ≥40% from baseline or the initiation of renal replacement therapy (dialysis or kidney transplantation) [20]. Time to the first qualifying event was used in the survival analyses. eGFR levels were obtained from electronic medical records during follow-up. During the follow-up period, no deaths were identified in the available medical records of the study cohort. Therefore, competing risk analysis for mortality was not performed.

### 2.5. Statistical Analysis

Data were summarized as percentages for categorical variables and means ± standard deviation (SD) or median (interquartile range) for continuous variables. Skewed variable (proteinuria) underwent a natural logarithmic transformation before the analysis. Categorical variables were analyzed using a chi-squared test, whereas continuous variables were compared using Student’s *t*-test or a Mann–Whitney U test, as appropriate. *p* for trend was calculated by the Cochran–Armitage trend test or the Jonckheere–Terpstra test for the distribution of each clinical parameter, stratified by cortical IFTA score. Kidney survival was estimated using the Kaplan–Meier method and compared by the log-rank test. Patients were censored at the last follow-up or 31 October 2024. Cox proportional hazards models were used to estimate hazard ratios (HRs) and 95% confidence intervals (CIs), with adjustment for covariates selected based on biological plausibility and prior reports. The proportional hazards assumption was assessed using scaled Schoenfeld residuals. Discriminative ability was assessed by Harrell’s concordance index (C-index) [21]. Correlations between urine pH and clinical variables were evaluated using Spearman’s correlation analysis. To evaluate factors associated with urine pH, we performed ordinal logistic regression analyses using categorized urine pH levels, and multiple linear regression analyses treating urine pH as a continuous variable. Candidate variables included age, sex, eGFR, diabetes, hypertension, BMI, urinary protein levels, ACE-I or ARB use, and hematuria. The proportional-odds assumption for ordinal logistic regression was assessed using the Brant test. Statistical analyses were performed using JMP version 17.0.0 (SAS Institute, Inc., Cary, NC, with auxiliary computations for the C-index and spline modeling conducted in EZR (Easy R, Saitama Medical Center, Jichi Medical University, Saitama, Japan), a graphical user interface for R.). All tests were two-sided, and *p* < 0.05 was considered statistically significant. Complete data were available for all variables included in the main analyses. Therefore, no missing-data imputation was performed, and all 503 patients were included in the analyses.

## 3. Results

### 3.1. Patient Characteristics at the Time of Kidney Biopsy

Among 1167 patients who underwent a kidney biopsy, 503 patients met the selection criteria and were enrolled in the analysis (Figure 1). Baseline characteristics of included and excluded patients are compared (Supplementary Table S1). Clinical and histopathological variables at the time of kidney biopsy, stratified by urine pH and cortical IFTA score, as well as primary clinicopathologic diagnoses were summarized (Table 1, Supplemental Tables S2 and S3). The mean age was 54.3 ± 17.7 years; 222 patients (44%) were male, 126 patients (25%) had a history of diabetes, 235 patients (46.7%) had a history of hypertension, the mean eGFR was 60.7 ± 26.2 mL/min per 1.73 m^2^, and urine pH was 6.15 ± 0.84, and median proteinuria was 1.1 g/gCr (interquartile range (IQR), 0.37–3.1). Patients with lower urine pH had higher serum creatinine levels, higher cortical IFTA scores, more severe medullary fibrosis and cast formation, and a higher prevalence of hypertension and diabetes. They also had lower eGFR and BMI.

### 3.2. Prognostic Significance of Histopathologic Lesions

**Figure 3:**
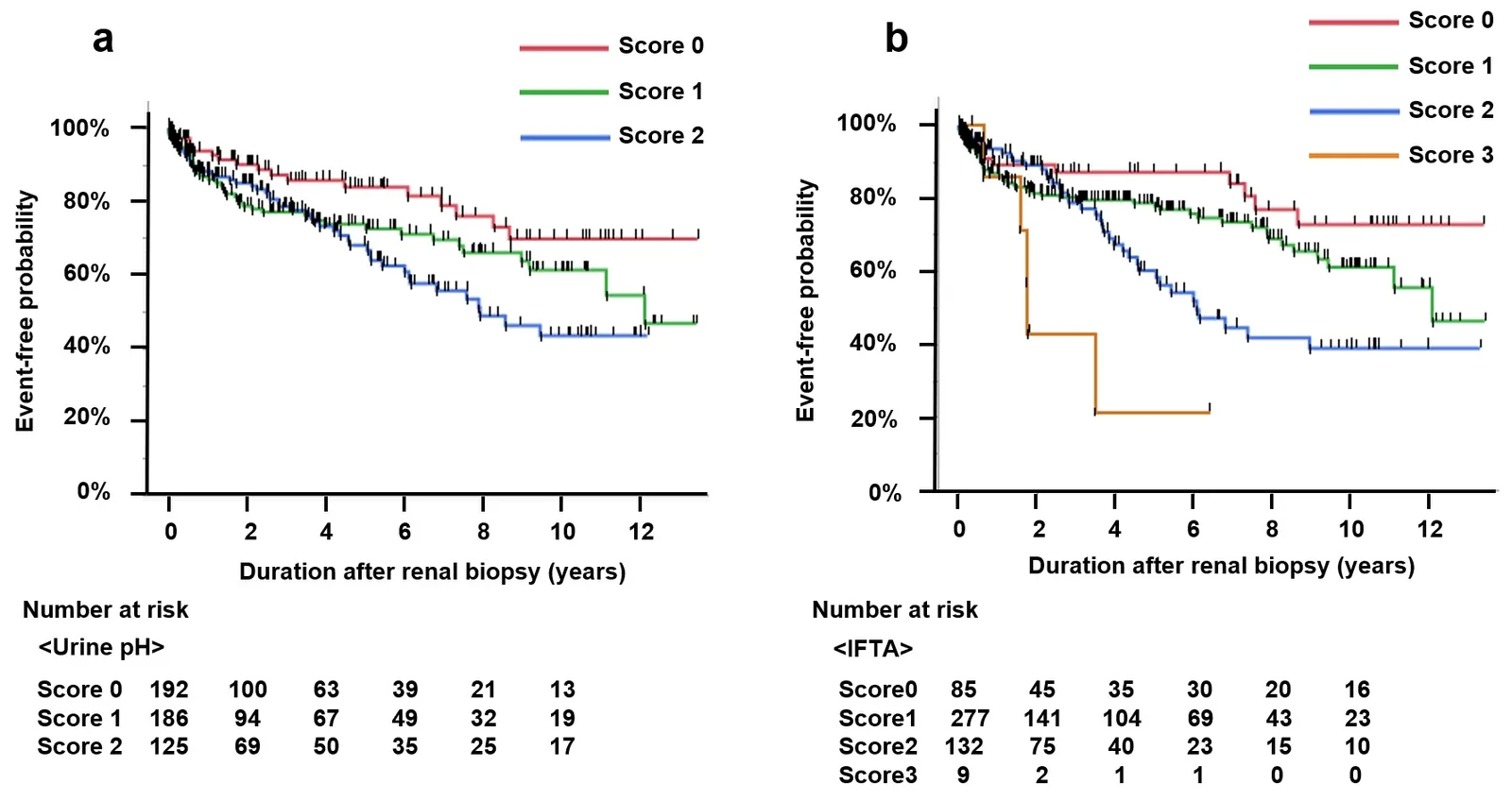
Renal survival rate stratified by urine pH and cortical IFTA score. (**a**) Renal survival rate stratified by urine pH score: score 0 (pH < 6.0), score 1 (pH 6.0–<7.0), and score 2 (pH ≥ 7.0). Log-rank test: *p* = 0.019. (**b**) Renal survival rate stratified by cortical IFTA score. Log-rank test: *p* < 0.001. IFTA, interstitial fibrosis and tubular atrophy.

**Figure 4:**
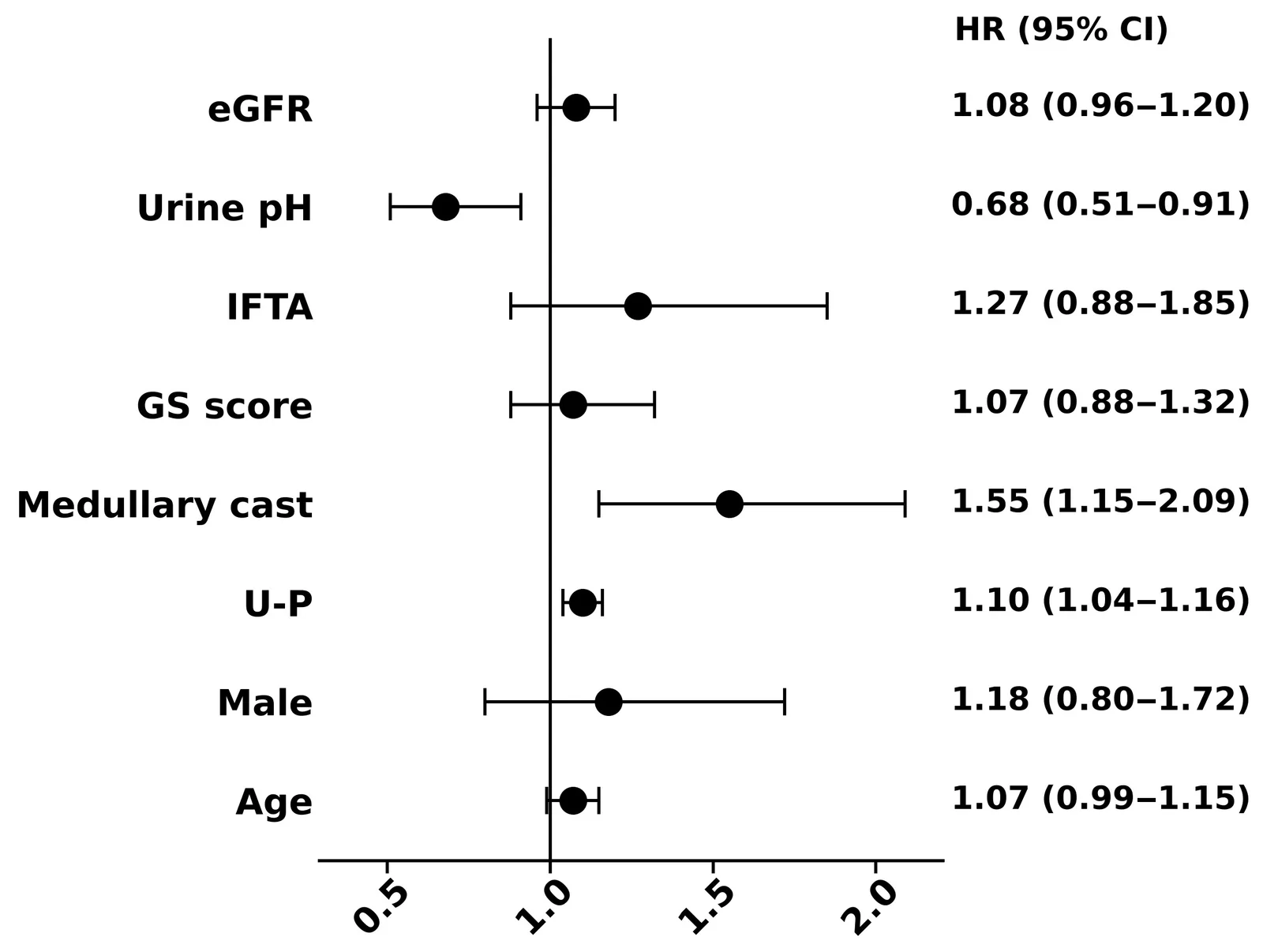
Multivariable Cox proportional hazards models for kidney outcomes. Forest plot showing hazard ratios and 95% confidence intervals for the associations of urine pH, eGFR (per 10 mL/min/1.73 m^2^ increase), cortical interstitial fibrosis and tubular atrophy (IFTA) score, global glomerulosclerosis (GS) score, medullary cast formation score, urine protein (U-P), age (per 5-year increase), and sex with kidney outcomes.

During the follow-up period, 113 patients reached the primary endpoint. All 113 first qualifying events were attributable to an eGFR decline of ≥40%, and no patient initiated renal replacement therapy before reaching this threshold. Therefore, the observed associations in the Cox proportional hazards analyses were driven entirely by events defined as an eGFR decline of ≥40%. The Kaplan–Meier curves showed early separation during follow-up. Kaplan–Meier analyses showed that higher cortical IFTA scores were associated with an increased risk of kidney outcomes. Similarly, compared with the intermediate urine pH group, kidney survival was lowest in the low urine pH group and highest in the high urine pH group (Figure 3). In Cox proportional hazards models adjusted for age, gender, eGFR and proteinuria, both urine pH categories and cortical IFTA scores were independently associated with kidney outcomes (Table 2, Figure 4). This association remained significant after further adjustment for cortical IFTA score (hazard ratio [HR], 0.71; 95% confidence interval [CI], 0.54–0.95). Although urine pH remained associated with kidney outcomes after adjustment for cortical IFTA, interpretation of findings in the cortical IFTA grade 3 subgroup should be cautious because of the limited sample size. Although urine pH was measured semi-quantitatively using dipstick testing, analyses treating urine pH as either categorical or continuous yielded similar results (HR, 0.69; 95% CI, 0.52–0.92) (Table 2). The proportional hazards assumption was assessed using Schoenfeld residuals. No significant violation was observed for urine pH across all models (all *p* > 0.05). Although the global tests indicated violations in Models 2–4, these were mainly attributable to age. Additional analyses using urine pH score categories showed similar overall trends, although statistical significance was attenuated after multivariable adjustment (Supplemental Table S4).

To assess whether medullary pathological findings improved prognostic discrimination beyond conventional risk factors, the C-index was calculated. A model including age, sex, eGFR, and proteinuria yielded a C-index of 0.632 (standard error [SE], 0.030). Addition of cortical IFTA increased the C-index to 0.642 (SE, 0.028), and further inclusion of urine pH increased it to 0.654 (SE, 0.028), indicating incremental improvement in prognostic performance. Although the increase in C-index was numerically small, addition of urine pH significantly improved model fit (likelihood ratio test, *p* = 0.009).

In a subgroup analysis of patients with IgA nephropathy (*n* = 158), 28 kidney outcome events occurred during a median follow-up of 3.1 years. Kaplan–Meier analysis showed a generally similar pattern to that observed in the overall cohort; however, the differences were not statistically significant (Supplemental Figure S1). Because of the limited number of events and potential lack of statistical power, these findings should be considered exploratory and interpreted cautiously. Multivariable Cox analysis was not performed.

### 3.3. Clinical and Histopathological Factors Associated with Urine pH and Medullary Cast Formation

To explore predictors of lower urine pH, patients were stratified according to the underlying kidney disease. Mean urine pH varied across disease categories, with relatively higher values observed in minimal change disease and membranous nephropathy, and lower values in diabetic nephropathy and nephrosclerosis, indicating heterogeneity across disease etiologies (Supplemental Figure S2). Next, factors associated with urine pH were evaluated using Spearman’s correlation and ordinal logistic regression analyses (Table 3 and Supplemental Table S5). Urine pH was positively correlated with eGFR (Spearman’s ρ = 0.30, *p* < 0.001) and inversely correlated with medullary cast score (ρ = −0.19, *p* < 0.001) and cortical IFTA score (ρ = −0.25, *p* < 0.001). Correlations between urine pH and clinical variables are shown in Supplemental Figure S3. In ordinal logistic regression analysis, lower eGFR, higher urine protein, and cortical IFTA were significantly associated with lower urine pH. Male sex was also associated with urine pH, although the effect size was modest. Age was not significantly associated.

We further examined factors associated with medullary cast formation. Spearman’s correlation analysis showed that medullary cast formation score was positively correlated with GS score (ρ = 0.34, *p* < 0.001), cortical IFTA score (ρ = 0.49, *p* < 0.001), and medullary fibrosis score (ρ = 0.54, *p* < 0.001), and inversely correlated with eGFR (ρ = −0.48, *p* < 0.001) and urine pH score (ρ = −0.19, *p* < 0.001) (Supplemental Table S6). In multivariable ordinal logistic regression analysis, GS score showed a positive but statistically nonsignificant association with medullary cast formation (odds ratio [OR], 1.21; 95% confidence interval [CI], 0.98–1.49; *p* = 0.070). The association between urine pH and medullary cast formation was also not statistically significant after adjustment for clinical and histopathological factors (OR, 0.85; 95% CI, 0.65–1.10; *p* = 0.21). In contrast, cortical IFTA (OR, 1.92; 95% CI, 1.29–2.86; *p* = 0.001) and medullary fibrosis (OR, 3.02; 95% CI, 2.20–4.16; *p* < 0.001) remained significantly associated with medullary cast formation (Supplemental Table S7).

## 4. Discussion

In this biopsy-proven kidney disease cohort, lower urine pH was independently associated with adverse kidney outcomes, even after adjustment for established prognostic factors, including baseline eGFR, proteinuria, and cortical IFTA score. In addition, lower urine pH was associated with greater medullary cast formation in unadjusted analyses, although this association was attenuated after multivariable adjustment. These findings suggest that urine pH may serve as a clinical marker associated with kidney prognosis and tubular pathological changes, without establishing a direct causal relationship. Although the addition of urine pH improved model fit, as indicated by the likelihood ratio test, the increase in the C-index was modest (from 0.642 to 0.654). Therefore, the incremental predictive value of urine pH may be limited, and the clinical significance of this improvement remains uncertain. Further studies incorporating external validation are needed to determine whether urine pH provides clinically meaningful improvements in risk prediction.

Urine pH is influenced by renal hydrogen ion handling and urinary buffering processes, but does not directly quantify net renal acid excretion [9]. Previous studies have reported that lower urine pH is associated with adverse kidney outcomes, including CKD progression, across diverse populations [12,13,14,15,16]. Our findings extend these observations to a biopsy-based cohort, enabling direct evaluation of histopathological correlates. Importantly, urine pH retained prognostic significance even after adjustment for cortical IFTA, suggesting that it captures information beyond static structural injury and may reflect ongoing tubular stress or maladaptive responses. Although urine dipstick testing is widely used in routine clinical practice because of its simplicity, accessibility, and low cost, its semi-quantitative nature limits measurement precision. More accurate methods, such as calibrated glass electrode-based pH meters, allow urine pH to be assessed as a continuous numerical variable and may provide a more precise characterization of its association with kidney outcomes. Furthermore, repeated measurements using standardized urine collection and analysis procedures could account for intraindividual variability related to dietary intake, circadian variation, and other physiological factors. Future prospective studies incorporating serial, electrode-based urine pH measurements are warranted to validate our findings and determine whether longitudinal changes in urine pH provide additional prognostic information beyond a single measurement.

Experimental and clinical studies provide mechanistic support for a role of urinary acid–base status in tubular injury [6,10,11]. Dietary alkali intake and alkali supplementation have been associated with slower CKD progression and limitation of oxidative stress, potentially through improvement in the intratubular environment [22,23], although these mechanisms remain incompletely understood. Urine pH is influenced by multiple physiological and clinical factors, including dietary acid load, muscle mass, renal ammonium excretion, and kidney function. Therefore, lower urine pH does not necessarily indicate greater net acid excretion or a more acidic intrarenal environment. Although we adjusted for established prognostic factors, residual confounding by unmeasured factors affecting urinary acid–base regulation cannot be excluded. Accordingly, the observed associations should be interpreted cautiously and should not be considered evidence of a direct causal relationship. Differences in urine pH among disease categories may partly reflect variations in kidney function rather than disease-specific mechanisms. In particular, the lower urine pH observed in diabetic nephropathy and nephrosclerosis may have been influenced by lower eGFR levels in these populations. Furthermore, because the study cohort included heterogeneous kidney diseases with differing degrees of proteinuria and tubulointerstitial injury, the mechanisms linking urine pH, medullary cast formation, and kidney outcomes may not be uniform across disease categories. Medullary cast formation may also depend on disease-specific factors, including urinary protein handling and interactions with uromodulin.

**Figure 5:**
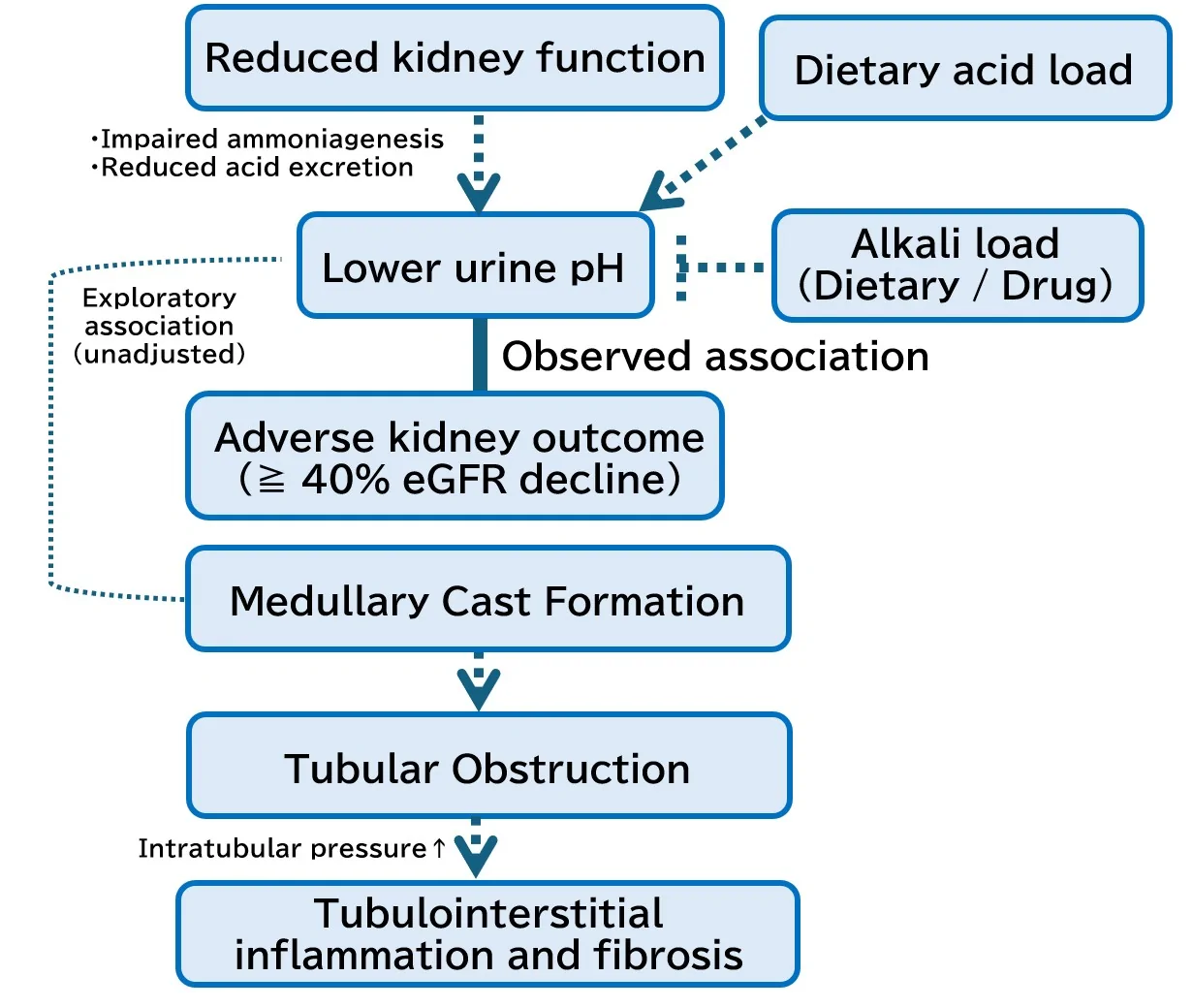
Observed associations and hypothesized mechanisms involving urine pH, medullary cast formation, and kidney outcomes. Solid lines indicate associations observed in the present study, whereas dotted lines represent exploratory associations observed in unadjusted analyses. Dashed arrows indicate hypothesized pathophysiological mechanisms that were not directly established in this study. Lower urine pH was independently associated with adverse kidney outcomes, whereas its association with medullary cast formation was attenuated after multivariable adjustment. The potential contribution of medullary cast formation to tubular obstruction and subsequent tubulointerstitial inflammation and fibrosis remains hypothetical. None of the depicted relationships establish causality.

An exploratory finding of this study was the association between lower urine pH and greater medullary cast formation in unadjusted analyses. However, this association was attenuated after adjustment for clinical and pathological factors, suggesting that it may largely reflect shared associations with kidney dysfunction and tubulointerstitial injury rather than an independent effect of urine pH on cast formation. Acidic tubular conditions may reduce the solubility of filtered proteins and promote aggregation within the medullary environment, potentially contributing to tubular obstruction. Indeed, experimental study supports this concept [24], demonstrating that alkalinization reduces tubular cast formation and tubulointerstitial injury. On the other hand, the association between urine pH and medullary cast formation was attenuated after multivariable adjustment. This finding suggests that urine pH may not act as an independent determinant of cast formation, but rather reflects one aspect of a complex pathophysiological process involving reduced kidney function, tubular injury, and altered intrarenal microenvironment. Reduced kidney function may promote urinary acidification, while cast formation may further contribute to tubular injury and functional decline. Thus, urine pH may function as an integrative marker linking kidney function and tubular pathology (Figure 5). From a clinical perspective, urine pH is a simple, readily available, and repeatedly measurable clinical marker. Alkali supplementation and dietary interventions have been reported to slow CKD progression [22,25]. However, the present observational study cannot determine whether modifying urine pH would alter medullary cast formation or kidney outcomes. Further interventional and mechanistic studies are needed to investigate these possibilities. Although the observed association between lower urine pH and medullary cast formation is biologically plausible, the present observational study does not establish a direct mechanistic relationship. Therefore, urine pH may function as a marker of underlying tubular dysfunction and impaired renal acid–base handling rather than a direct causal mediator. Further mechanistic studies evaluating urinary ammonium excretion, tubular acid–base regulation, and protein handling under different urinary pH conditions are warranted.

This study has several limitations. First, this was a retrospective single-center study, which may limit generalizability and introduce selection bias. Furthermore, the cohort consisted exclusively of patients who underwent kidney biopsy at a Japanese tertiary referral center and had evaluable medullary tissue. Therefore, the findings may not be generalizable to the overall CKD population, particularly patients who do not undergo kidney biopsy. eGFR was calculated using the equation developed by the Japanese Society of Nephrology, which may limit the generalizability of the findings to non-Japanese populations. Although the number of events was considered acceptable for the primary multivariable analyses, subgroup analyses, particularly those involving patients with IgA nephropathy, may have been underpowered and should be interpreted cautiously. In addition, because separation of the Kaplan–Meier curves was more prominent during the early follow-up period, the proportional hazards assumption may not have been fully satisfied. Second, only 503 of the 1167 patients who underwent kidney biopsy were included in the analysis. The requirement for evaluable medullary tissue and other eligibility criteria may have introduced selection bias, potentially limiting the generalizability of our findings. Furthermore, the study population included patients with heterogeneous underlying kidney diseases. Differences in disease-specific pathophysiology, urine acidification, and rates of kidney function decline may have influenced the observed association between urine pH and kidney outcomes. Although subgroup analysis of patients with IgA nephropathy showed a generally similar trend, the association was not statistically significant, possibly due to the limited number of events. Therefore, residual confounding by underlying kidney disease cannot be excluded. Further studies with larger disease-specific cohorts are warranted to determine whether the prognostic association of urine pH is consistent across different kidney diseases. Third, urine pH was measured semi-quantitatively using a dipstick test at a single time point and may have been influenced by dietary factors, medications, and pre-analytical factors such as specimen handling, standing time, temperature, and bacterial contamination, potentially introducing measurement imprecision and exposure misclassification. Although dipstick testing is widely used in routine clinical practice, its limited measurement precision and the absence of repeated measurements may have affected the observed associations. Furthermore, validation using more precise, electrode-based pH measurements was not performed in the present study. Future prospective studies incorporating repeated urine pH measurements using a calibrated pH meter are warranted to confirm our findings and assess urine pH as a continuous numerical variable. Information on urinary tract infections, bacteriuria, and the use of medications affecting urine pH, including alkali supplementation, was not systematically available for all patients. Therefore, these factors could not be comprehensively accounted for in the analyses and may have contributed to residual confounding. Fourth, urinary ammonium excretion and serum bicarbonate levels were not assessed, precluding a more comprehensive evaluation of renal acid–base handling. Therefore, lower urine pH may partly reflect systemic metabolic acidosis rather than a specific independent intrarenal process. In addition, detailed dietary data, including protein intake, fruit and vegetable consumption, and alkali supplementation, were not available. Therefore, the proposed relationships between dietary acid load and urine pH in the conceptual framework remain hypothetical. Fifth, biopsy sampling may not fully capture medullary pathology because of anatomical heterogeneity and limited tissue availability. In addition, medullary pathological findings were assessed jointly by two nephrologists using consensus scoring, and interobserver agreement could not be independently evaluated. Finally, as an observational study, causal relationships cannot be established.

## 5. Conclusions

In conclusion, lower urine pH was independently associated with adverse kidney outcomes in patients undergoing native kidney biopsy. Lower urine pH was also associated with greater medullary cast formation in unadjusted analyses, although this association was attenuated after multivariable adjustment. These findings suggest that urine pH may serve as a readily available clinical marker associated with kidney prognosis. Further prospective and mechanistic studies are needed to clarify the underlying relationships and determine whether changes in urine pH influence kidney outcomes.

## Figures and Tables

**Table 1:** Baseline clinical and histopathological characteristics according to urine pH score.

		All Patients (*n* = 503)	Urine pH Score			*p* for Trend
			0 (*n* = 192)	1 (*n* = 186)	2 (*n* = 125)	
Men		222 (44.0%)	95 (49.5%)	84 (45.2%)	44 (35.2%)	0.016
Age, years		54.3 ± 17.7	56.9 ± 16.2	54.2 ± 18.8	50.2 ± 17.9	0.003
BMI, kg/m^2^		22.7 ± 4.0	23.1 ± 4.3	22.9 ± 3.7	21.8 ± 3.7	0.009
sBP, mmHg		127.3 ± 18.6	128.7 ± 19.2	128.7 ± 17.6	123.2 ± 18.6	0.015
dBP, mmHg		79.4 ± 12.5	80.1 ± 13.0	78.9 ± 11.7	78.8 ± 12.8	0.41
sCr, mg/dL		1.06 ± 0.49	1.21 ± 0.56	1.07 ± 0.46	0.83 ± 0.32	<0.001
eGFR, mL/min/1.73 m^2^		60.7 ± 26.2	52.9 ± 22.8	60.2 ± 26.8	73.9 ± 25.5	<0.001
HbA1c, %		5.7 (5.4–6.1)	5.8 (5.5–6.2)	5.7 (5.4–6.2)	5.6 (5.3–5.9)	0.001
U-P, g/gCr		1.1 (0.37–3.1)	0.98 (0.37–2.1)	1.4 (0.44–4.0)	1.0 (0.31–3.4)	0.21
Hematuria, %		296 (58.8%)	113 (58.9%)	113 (60.8%)	70 (56.0%)	0.71
Urine pH		6.15 ± 0.84	5.40 ± 0.20	6.20 ± 0.25	7.36 ± 0.44	<0.001
Hypertension		235 (46.7%)	102 (53.1%)	89 (47.8%)	44 (35.2%)	0.003
DM		126 (25.0%)	54 (28.1%)	51 (27.4%)	21 (16.8%)	0.040
ACE-I or ARB		175 (34.8%)	87 (45.3%)	62 (33.3%)	26 (20.8%)	<0.001
Ca blocker		176 (35.0%)	74 (38.5%)	66 (35.5%)	36 (28.8%)	0.087
Statins		121 (24.1%)	47 (24.5%)	44 (23.7%)	30 (24.0%)	0.87
cortical IFTA	0	85 (16.9%)	20 (10.4%)	27 (14.5%)	38 (30.4%)	<0.001
	1	277 (55.0%)	97 (50.5%)	111 (59.7%)	69 (55.2%)	
	2	132 (26.2%)	73 (38.0%)	42 (22.6%)	17 (13.6%)	
	3	9 (1.8%)	2 (1.0%)	6 (3.2%)	1 (0.8%)	
Global glomerulosclerosis score	0	212 (42.1%)	76 (39.6%)	72 (38.7%)	64 (51.2%)	0.024
	1	129 (25.6%)	45 (23.4%)	52 (28.0%)	32 (25.6%)	
	2	108 (21.5%)	49 (25.5%)	39 (21.0%)	20 (16.0%)	
	3	54 (10.7%)	22 (11.5%)	23 (12.4%)	9 (7.2%)	
Medullary fibrosis	0	138 (27.4%)	43 (22.4%)	53 (28.5%)	42 (33.6%)	0.001
	1	248 (49.3%)	94 (49.0%)	86 (46.2%)	68 (54.4%)	
	2	117 (23.3%)	55 (28.6%)	47 (25.3%)	15 (12.0%)	
Medullary inflammatory cell infiltration	0	367 (72.9%)	131 (68.2%)	137 (73.7%)	99 (79.2%)	0.049
	1	111 (22.1%)	54 (28.1%)	36 (19.4%)	21 (16.8%)	
	2	25 (5.0%)	7 (3.6%)	13 (7.0%)	5 (4.0%)	
Medullary cast formation	0	259 (51.5%)	80 (41.7%)	95 (51.1%)	84 (67.2%)	<0.001
	1	178 (35.4%)	80 (41.7%)	66 (35.5%)	32 (25.6%)	
	2	66 (13.1%)	32 (16.7%)	25 (13.4%)	9 (7.2%)	

Values reported as n (%) for categorical variables and mean ± standard deviation or median (interquartile range) for continuous variables. BMI, body mass index; sBP, systolic blood pressure; dBP, diastolic blood pressure; sCr, serum creatinine; eGFR, estimated glomerular filtration rate; HbA1c, hemoglobin A1c; U-P, urinary protein excretion; DM, diabetes mellitus; ACE-I, angiotensin-converting enzyme inhibitor; ARB, angiotensin II type 1 receptor blocker; IFTA, interstitial fibrosis and tubular atrophy. Urine pH scores were defined as follows: score 0, pH < 6.0; score 1, pH 6.0–<7.0; and score 2, pH ≥ 7.0. The values 5.40, 6.20, and 7.36 represent the mean measured urine pH within each respective category, rather than the score definitions.

**Table 2:** Multivariable Cox proportional hazards models for kidney outcomes.

	Model 0 HR (95%CI)	Model 1 HR (95%CI)	Model 2 HR (95%CI)	Model 3 HR (95%CI)	Model 4 HR (95%CI)
Urine pH score (per score)	0.70 (0.55–0.90)	0.76 (0.59–0.98)	0.69 (0.52–0.91)	0.71 (0.54–0.95)	0.68 (0.51–0.90)
Urine pH (continuous)	0.68 (0.53–0.87)	0.73 (0.56–0.94)	0.67 (0.50–0.89)	0.69 (0.52–0.92)	0.67 (0.51–0.90)

Model 0: No adjustment factors. Model 1: Sex, Age. Model 2: Model 1 + Urinary protein, eGFR, systolic blood pressure. Model 3: Model 2 + cortical IFTA. Model 4: Model 2 + medullary cast formation. HR, hazard ratio; 95%CI, 95% confidence interval; IFTA, interstitial fibrosis and tubular atrophy. Schoenfeld residual-based tests showed no significant violation of the proportional hazard assumption for urine pH in any model (all *p* > 0.05), although the global tests were significant in some adjusted models.

**Table 3:** Ordinal logistic regression analysis by urine pH score.

	Urine pH Score	
	Estimated Odds (95%CI)	*p*-Value
Sex (male)	0.71 (0.50–1.00)	0.048
Age (increased by 5 years)	1.03 (0.97–1.09)	0.38
eGFR (decreased by 10 mL/min/1.73 m^2^)	1.21 (1.10–1.33)	<0.001
U-P	1.08 (1.03–1.14)	0.004
Hypertension	0.88 (0.60–1.29)	0.51
DM	0.73 (0.49–1.09)	0.12
cortical IFTA	0.67 (0.48–0.94)	0.020
Global glomerulosclerosis score	1.13 (0.93–1.36)	0.22
Medullary fibrosis	1.19 (0.90–1.58)	0.22
Medullary cast formation	0.81 (0.60–1.09)	0.16

95%CI, 95% confidence interval; eGFR, estimated glomerular filtration rate; U-P, urinary protein excretion; DM, diabetes mellitus. The proportional-odds assumption was assessed using the Brant test. No significant violation was detected in the omnibus test (χ^2^ = 11.66, df = 10, *p* = 0.308), although a significant violation was observed for U-P (*p* = 0.009).

## Data Availability

The data that support the findings of this study are available from the corresponding author upon reasonable request.

## Supplementary Materials

The following supporting information can be downloaded at https://www.mdpi.com/article/10.3390/diagnostics16203418/s1. Supplemental Table S1. Comparison of patients with adequate and insufficient medullary tissue. Supplemental Table S2. Clinical and histopathological findings for all patients and for patients stratified by cortical IFTA score. Supplemental Table S3. Clinicopathologic diagnoses of the study population. Supplemental Table S4. Cox proportional hazards analysis of urine pH score. Supplemental Table S5. Spearman’s correlation coefficients among clinical and pathological variables in urine pH score. Supplemental Table S6. Spearman’s correlation coefficients among clinical and pathological variables in medullary cast formation score. Supplemental Table S7. Ordinal logistic regression analysis by medullary cast score. Supplemental Figure S1. Renal survival rate stratified by urine pH and cortical IFTA score in IgA nephropathy. Supplemental Figure S2. Urine pH according to underlying kidney disease. Supplemental Figure S3. Correlation between urine pH and clinical variables; STROBE Statement.

## Abbreviations

The following abbreviations are used in this manuscript:

IFTA	interstitial fibrosis and tubular atrophy
CKD	Chronic kidney disease
eGFR	estimated glomerular filtration rate
BMI	body mass index
HbA1c	hemoglobin A1c
ACE-I	angiotensin-converting enzyme inhibitor
ARB	angiotensin II receptor blocker
