# Supplementary material for: Association Between Urine pH and Kidney Outcomes in Patients with Biopsy-Proven Kidney Disease
Source: Diagnostics (Basel). 2026 Oct 20;16(20):3418. doi: 10.3390/diagnostics16203418 (PMC13703313; doi:10.3390/diagnostics16203418)
Supplement: Supplementary file 1 [file diagnostics-16-03418-s001.zip › diagnostics-4520343-supplementary.pdf]

# Supplemental Material

## Table of contents

**1. Supplemental Table**

**Supplemental Table S1.** Comparison of patients with adequate and insufficient medullary tissue

**Supplemental Table S2.** Clinical and histopathological findings for all patients and for patients stratified by IFTA score

**Supplemental Table S3.** Clinicopathologic diagnoses of the study population

**Supplemental Table S4.** Cox proportional hazards analysis of urine pH score

**Supplemental Table S5.** Spearman's correlation coefficients among clinical and pathological variables in urine pH score

**Supplemental Table S6.** Spearman's correlation coefficients among clinical and pathological variables in medullary cast formation score

**Supplemental Table S7.** Ordinal logistic regression analysis by medullary cast score

**2. Supplemental Figure**

**Supplemental Figure S1.** Renal survival rate stratified by urine pH and cortical IFTA score in IgA nephropathy.

**Supplemental Figure S2.** Urine pH according to underlying kidney disease.

**Supplemental Figure S3.** Correlation between urine pH and clinical variables.

**3. STROBE Statement**

**Supplemental Table S1. Comparison of patients with adequate and insufficient medullary tissue**

| | Medullary tissue | | P value |
|---|---|---|---|
| | adequate (n=503) | insufficient (n=587) | |
| Men | 223 (44.3%) | 322 (54.9%) | <0.001 |
| Age, years | 54.2±17.8 | 51.5±17.6 | 0.011 |
| BMI, $kg/m^2$ | 22.7±4.0 | 23.4±3.9 | 0.002 |
| sBP, mmHg | 127.3±18.6 | 131.9±17.7 | <0.001 |
| dBP, mmHg | 79.3±12.5 | 81.2±12.1 | 0.014 |
| sCr, mg/dL | 1.06±0.50 | 1.05±0.50 | 0.78 |
| eGFR, mL/min/1.73 $m^2$ | 60.8±26.3 | 64.3±28.3 | 0.036 |
| U-P, g/gCr | 1.12 (0.37–3.09) | 1.00 (0.39–3.30) | 0.64 |

Data are presented as n (%), mean ± SD, or median (interquartile range). P values were calculated using the chi-square test for categorical variables, the unpaired Student's t-test for normally distributed continuous variables, and the Mann–Whitney U test for U-P. BMI, body mass index; sBP, systolic blood pressure; dBP, diastolic blood pressure; sCr, serum creatinine; eGFR, estimated glomerular filtration rate; U-P, urinary protein excretion. Patients aged <18 years or with eGFR <15 mL/min/1.73 $m^2$ were excluded.

**Supplemental Table S2. Clinical and histopathological findings for all patients and for patients stratified by cortical IFTA score**

| | | All patients (n=503) | IFTA score | | | | p for trend |
|---|---|---|---|---|---|---|---|
| | | | 0 (n=85) | 1 (n=277) | 2 (n=132) | 3 (n=9) | |
| Men | | 222 (44.0%) | 37 (43.5%) | 129 (46.6%) | 52 (39.4%) | 5 (55.6%) | 0.52 |
| Age, years | | 54.3±17.7 | 41.6±18.0 | 54.9±17.0 | 60.8±14.9 | 55.6±18.0 | <0.001 |
| BMI, kg/m$^2$ | | 22.7±4.0 | 22.9±4.2 | 22.7±3.7 | 22.4±4.5 | 23.5±3.9 | 0.58 |
| sBP, mmHg | | 127.3±18.6 | 120.4±15.8 | 127.0±18.9 | 131.9±18.3 | 135.7±17.9 | <0.001 |
| dBP, mmHg | | 79.4±12.5 | 77.5±10.5 | 79.5±12.1 | 80.3±14.1 | 75.9±15.6 | 0.28 |
| sCr, mg/dL | | 1.06±0.49 | 0.78±0.27 | 0.96±0.40 | 1.41±0.55 | 1.90±0.50 | <0.001 |
| eGFR, ml/min/1.73 m$^2$ | | 60.7±26.2 | 84.6±26.5 | 63.9±22.2 | 41.2±17.8 | 28.9±7.66 | <0.001 |
| HbA1c, % | | 5.7 (5.4-6.1) | 5.5 (5.3-5.8) | 5.8 (5.5-6.2) | 5.8 (5.4-6.1) | 6.1 (5.1-6.9) | 0.007 |
| U-P, g/gCr | | 1.10 (0.37-3.10) | 0.70 (0.16-3.50) | 1.00 (0.37-2.88) | 1.33 (0.57-3.04) | 4.99 (2.74-5.71) | 0.004 |
| Hematuria, % | | 296 (58.8%) | 44 (51.8%) | 178 (64.3%) | 67 (50.8%) | 7 (77.8%) | 0.66 |
| Urine pH | | 6.15±0.84 | 6.59±0.91 | 6.20±0.77 | 5.91±0.55 | 6.22±0.62 | <0.001 |
| Urine cast | | 233 (46.3%) | 27 (31.8%) | 133 (48.0%) | 68 (51.5%) | 6 (66.7%) | 0.005 |
| Hypertension | | 235 (46.7%) | 21 (24.7%) | 124 (44.8%) | 85 (64.4%) | 5 (55.6%) | <0.001 |
| DM | | 126 (25.0%) | 16 (18.8%) | 69 (24.9%) | 36 (27.3%) | 5 (55.6%) | 0.069 |
| ACE-I or ARB | | 175 (34.8%) | 11 (12.9%) | 103 (37.2%) | 58 (43.9%) | 3 (33.3%) | <0.001 |
| Ca blocker | | 176 (35.0%) | 16 (18.8%) | 87 (31.4%) | 67 (50.8%) | 6 (66.7%) | <0.001 |
| Statins | | 121 (24.1%) | 16 (18.8%) | 71 (25.6%) | 31 (23.5%) | 3 (33.3%) | 0.60 |
| Medullary fibrosis | 0 | 138 (27.4%) | 63 (74.1%) | 69 (24.9%) | 6 (4.5%) | 0 (0.0%) | <0.001 |
| | 1 | 247 (49.1%) | 20 (23.5%) | 164 (59.2%) | 63 (47.7%) | 1 (11.1%) | |
| | 2 | 117 (23.3%) | 2 (2.4%) | 44 (15.9%) | 63 (47.7%) | 8 (88.9%) | |
| Medullary inflammatory cell infiltration | 0 | 366 (72.8%) | 81 (95.3%) | 220 (79.4%) | 64 (48.5%) | 2 (22.2%) | <0.001 |
| | 1 | 111 (22.1%) | 4 (4.7%) | 54 (19.5%) | 51 (38.6%) | 2 (22.2%) | |
| | 2 | 25 (5.0%) | 0 (0.0%) | 3 (1.1%) | 17 (12.9%) | 5 (55.6%) | |
| Medullary cast formation | 0 | 258 (51.3%) | 78 (91.8%) | 153 (55.2%) | 27 (20.5%) | 1 (11.1%) | <0.001 |
| | 1 | 178 (35.4%) | 7 (8.2%) | 98 (35.4%) | 71 (53.8%) | 2 (22.2%) | |
| | 2 | 66 (13.1%) | 0 (0.0%) | 26 (9.4%) | 34 (25.8%) | 6 (66.7%) | |

Values reported as n (%) for categorical variables and mean ± standard deviation or median (interquartile range) for continuous variables. BMI, body mass index; sBP, systolic blood pressure; dBP, diastolic blood pressure; sCr, serum creatinine; eGFR, estimated glomerular filtration rate; HbA1c, hemoglobin A1c; U-P, urinary protein excretion; DM, diabetes mellitus; ACE-I, angiotensin–converting enzyme inhibitor; ARB, angiotensin II type 1 receptor blocker; IFTA, interstitial fibrosis and tubular atrophy.

**Supplemental Table S3. Clinicopathologic diagnoses of the study population**

| Immune-related glomerulopathy (n=319) | Non-immune-related glomerulopathy (n=98) | Metabolic diseases (n=54) | Tubular diseases (n=32) |
|---|---|---|---|
| IgA nephropathy (158) | MCNS (26) | Diabetic nephropathy (26) | TIN (28) |
| ANCA-associated vasculitis (50) | Minor glomerular changes (20) | Nephrosclerosis (24) | BK virus–associated nephropathy (1) |
| Lupus nephritis (37) | FSGS (19) | Obesity-related nephropathy (4) | Medullary cystic kidney disease (1) |
| MN (36) | TBM (14) | | Acute tubular necrosis (1) |
| IgA vasculitis (16) | Amyloidosis (8) | | Light-chain proximal tubulopathy (1) |
| MGPN (11) | TMA (4) | | |
| IRGN (3) | Alport syndrome (4) | | |
| Cryoglobulinemic vasculitis (3) | Fabry disease (2) | | |
| Immunotactoid nephropathy (2) | Lipoprotein glomerulopathy (1) | | |
| Non-IgA mesangial proliferative glomerulonephritis (1) | | | |
| Anti-GBM glomerulonephritis (1) | | | |
| Polyarteritis nodosa (1) | | | |

ANCA, anti-neutrophil cytoplasmic antibody; MN, membranous nephropathy; MPGN, membranoproliferative glomerulonephritis; IRGN, infection-related glomerulonephritis; GBM, glomerular basement membrane; MCNS, minimal change nephrotic syndrome; FSGS, focal segmental glomerulosclerosis; TBM, thin basement membrane disease; TMA, thrombotic microangiopathy; TIN, tubulointerstitial nephritis. TIN includes sarcoid nephropathy and IgG4-related kidney disease. TMA includes calcineurin inhibitor-associated nephropathy.

**Supplemental Table S4. Cox proportional hazards analysis of urine pH score.**

| | | Model 0<br>HR (95%CI) | Model 1<br>HR (95%CI) | Model 2<br>HR (95%CI) | Model 3<br>HR (95%CI) |
|---|---|---|---|---|---|
| Urine pH score | Score 0 | 1.28<br>(0.85-1.91) | 1.17<br>(0.78-1.76) | 1.36<br>(0.89-2.08) | 1.31<br>(0.86-2.01) |
| | Score 1 | Reference | Reference | Reference | Reference |
| | Score 2 | 0.60<br>(0.35-1.03) | 0.64<br>(0.37-1.11) | 0.61<br>(0.35-1.09) | 0.64<br>(0.36-1.14) |

Model 0: No adjustment factors. Model 1: Sex, Age (per 5 years). Model 2: Model 1 + Urinary protein, eGFR (per 10 mL/min/1.73m$^2$). Model 3: Model 2 + cortical IFTA. HR, hazard ratio; 95%CI, 95% confidence interval; IFTA, interstitial fibrosis and tubular atrophy.

**Supplemental Table S5. Spearman's correlation coefficients among clinical and pathological variables in urine pH score**

| | Urine pH score | |
|---|---|---|
| | Correlation coefficient | p-value |
| Age | -0.13 | 0.003 |
| BMI | -0.12 | 0.010 |
| sBP | -0.11 | 0.014 |
| dBP | -0.04 | 0.41 |
| eGFR | 0.30 | <0.001 |
| HbA1c | -0.14 | 0.001 |
| U-P | 0.06 | 0.22 |
| Global glomerulosclerosis score | −0.10 | 0.024 |
| cortical IFTA | -0.25 | <0.001 |
| Medullary fibrosis | -0.14 | 0.001 |
| Medullary inflammatory cell infiltration | -0.09 | 0.048 |
| Medullary cast formation | -0.19 | <0.001 |

BMI, body mass index; sBP, systolic blood pressure; dBP, diastolic blood pressure; eGFR, estimated glomerular filtration rate; HbA1c, hemoglobin A1c; U-P, urinary protein excretion; IFTA, interstitial fibrosis and tubular atrophy.

**Supplemental Table S6. Spearman's correlation coefficients among clinical and pathological variables in medullary cast formation score**

| | Medullary cast formation score | |
|---|---|---|
| | Correlation coefficient | p-value |
| Age | 0.30 | <0.001 |
| BMI | −0.04 | 0.41 |
| sBP | 0.14 | 0.002 |
| dBP | 0.01 | 0.78 |
| eGFR | −0.48 | <0.001 |
| HbA1c | 0.08 | 0.090 |
| U-P | 0.12 | 0.008 |
| cortical IFTA | 0.49 | <0.001 |
| Global glomerulosclerosis score | 0.34 | <0.001 |
| Medullary fibrosis | 0.54 | <0.001 |
| Medullary inflammatory cell infiltration | 0.31 | <0.001 |
| Urine pH score | −0.19 | <0.001 |

BMI, body mass index; sBP, systolic blood pressure; dBP, diastolic blood pressure; eGFR, estimated glomerular filtration rate; HbA1c, hemoglobin A1c; U-P, urinary protein excretion; IFTA, interstitial fibrosis and tubular atrophy.

**Supplemental Table S7. Ordinal logistic regression analysis by medullary cast formation score**

| | Medullary cast formation score | |
|---|---|---|
| | Estimated Odds (95%CI) | p-value |
| Urine pH score (per score) | 0.85 (0.65–1.10) | 0.21 |
| Sex (male) | 1.18 (0.80–1.75) | 0.40 |
| Age (increased by 5 years) | 1.04 (0.97–1.12) | 0.28 |
| eGFR (decreased by 10 ml/min/1.73 m²) | 0.84 (0.75–0.94) | 0.003 |
| U-P | 1.02 (0.96–1.09) | 0.55 |
| Hypertension | 0.86 (0.56–1.31) | 0.47 |
| DM | 0.61 (0.38–0.97) | 0.038 |
| cortical IFTA | 1.92 (1.29–2.86) | 0.001 |
| Global glomerulosclerosis score | 1.21 (0.98–1.49) | 0.070 |
| Medullary fibrosis | 3.02 (2.20–4.16) | <0.001 |

The proportional-odds assumption was assessed using the Brant test, with no significant violation in the omnibus test ($\chi^2 = 5.04$, df = 10, $p = 0.888$) or for any individual variable (all $p > 0.22$).

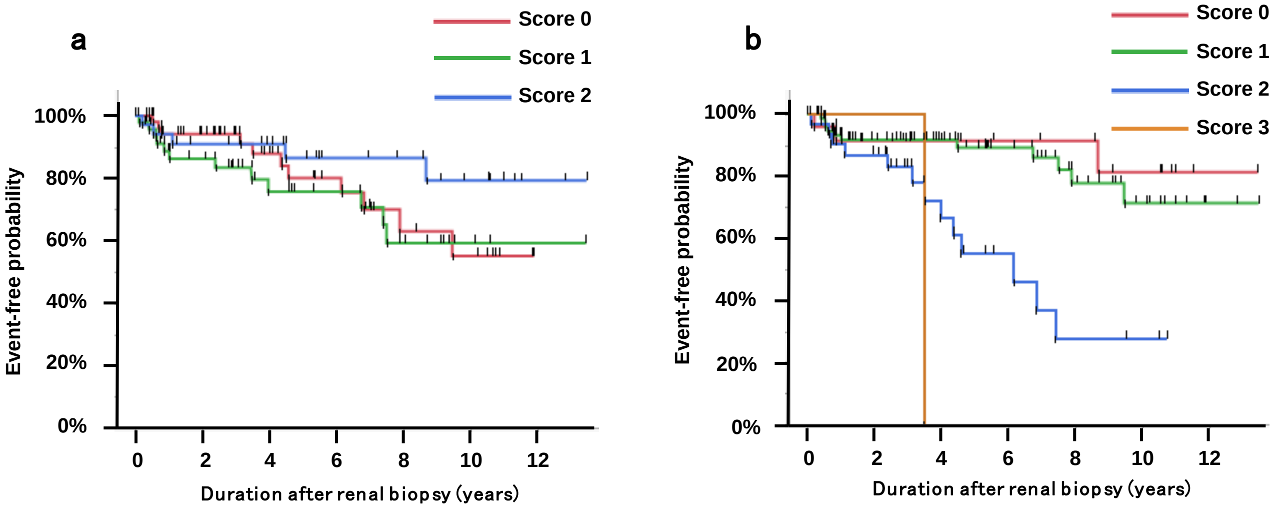


Number at risk

<Urine pH>

| | | | | | | |
|---|---|---|---|---|---|---|
| Score 0 | 68 | 40 | 26 | 17 | 9 | 7 |
| Score 1 | 50 | 33 | 19 | 15 | 9 | 3 |
| Score 2 | 40 | 27 | 23 | 15 | 13 | 9 |

Number at risk

<IFTA>

| | | | | | | |
|---|---|---|---|---|---|---|
| Score0 | 31 | 18 | 14 | 11 | 10 | 7 |
| Score1 | 90 | 56 | 42 | 30 | 18 | 10 |
| Score2 | 36 | 25 | 12 | 6 | 3 | 2 |
| Score3 | 1 | 1 | 0 | 0 | 0 | 0 |

**Supplemental Figure S1. Renal survival rate stratified by urine pH and cortical IFTA score in IgA nephropathy.**

(a) Renal survival rate stratified by urine pH score. Log rank test: $p = 0.241$. (b) Renal survival rate stratified IFTA score. Log rank test: $p < 0.001$. IFTA, interstitial fibrosis and tubular atrophy.

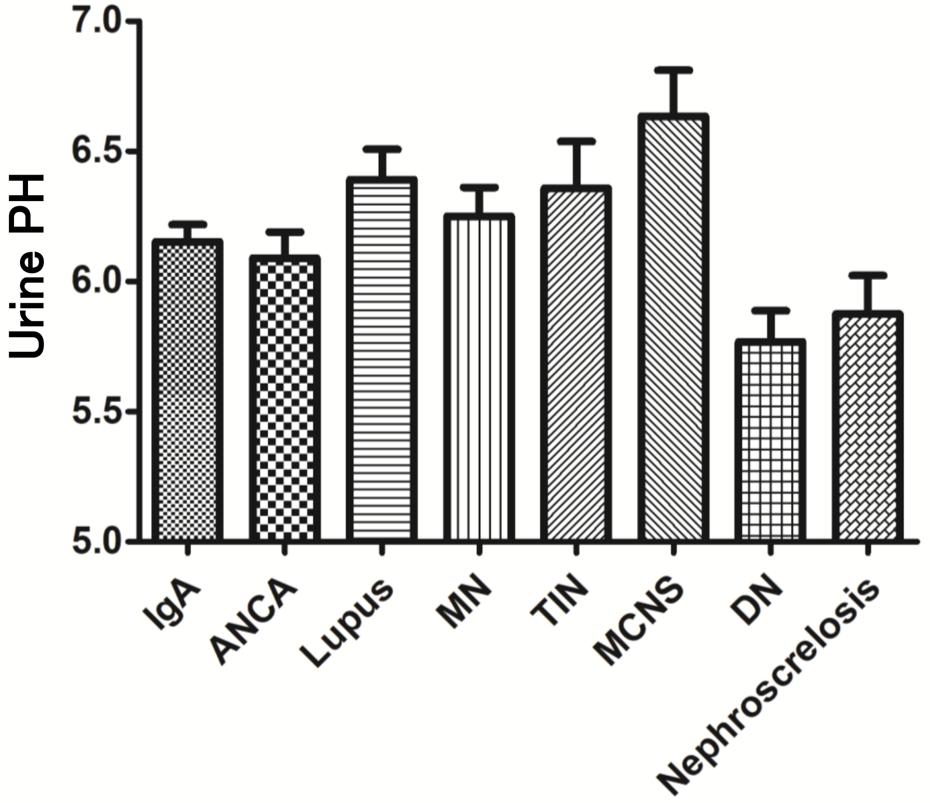


**Supplemental Figure S2. Urine pH according to underlying kidney disease.**

Urine pH according to underlying clinicopathological diagnoses in the study cohort. IgA nephropathy, ANCA-associated vasculitis, lupus nephritis, MN, TIN, MCNS, DN and nephrosclerosis. Boxes indicate median and interquartile range, with whiskers representing the range. ANCA, anti-neutrophil cytoplasmic antibody; MN, membranous nephropathy; TIN, tubulointerstitial nephritis; MCNS, minimal change nephrotic syndrome; DN, diabetic nephropathy.

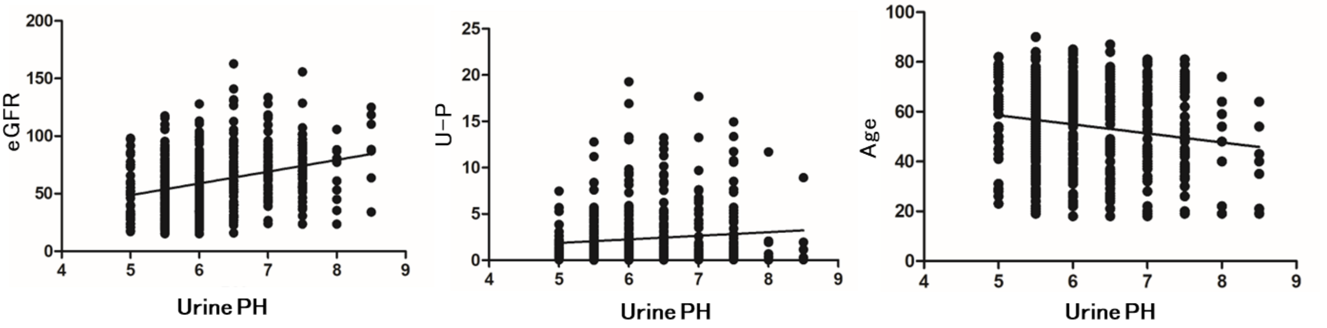


**Supplemental Figure S3. Correlation between urine pH and clinical variables.**
Scatter plots showing the relationships between urine pH and estimated glomerular filtration rate (eGFR), urine protein (U-P), and age at the time of kidney biopsy. Correlations were assessed using Spearman's rank correlation coefficient.

**STROBE Statement**

| | Item No | Recommendation | Page No |
|---|---|---|---|
| **Title and abstract** | 1 | (*a*) Indicate the study's design with a commonly used term in the title or the abstract | 1 |
| | | (*b*) Provide in the abstract an informative and balanced summary of what was done and what was found | 3-4 |
| **Introduction** | | | |
| Background/rationale | 2 | Explain the scientific background and rationale for the investigation being reported | 5-6 |
| Objectives | 3 | State specific objectives, including any prespecified hypotheses | 6 |
| **Methods** | | | |
| Study design | 4 | Present key elements of study design early in the paper | 7 |
| Setting | 5 | Describe the setting, locations, and relevant dates, including periods of recruitment, exposure, follow-up, and data collection | 7 |
| Participants | 6 | (*a*) Give the eligibility criteria, and the sources and methods of selection of participants. Describe methods of follow-up | 7 |
| | | (*b*) For matched studies, give matching criteria and number of exposed and unexposed | N/A |
| Variables | 7 | Clearly define all outcomes, exposures, predictors, potential confounders, and effect modifiers. Give diagnostic criteria, if applicable | 8-9 |
| Data sources/ measurement | 8* | For each variable of interest, give sources of data and details of methods of assessment (measurement). Describe comparability of assessment methods if there is more than one group | 8-9 |
| Bias | 9 | Describe any efforts to address potential sources of bias | 7-9 |
| Study size | 10 | Explain how the study size was arrived at | 7 |

| | | | |
|---|---|---|---|
| Quantitative variables | 11 | Explain how quantitative variables were handled in the analyses. If applicable, describe which groupings were chosen and why | 9-10 |
| Statistical methods | 12 | (*a*) Describe all statistical methods, including those used to control for confounding | 9-10 |
| | | (*b*) Describe any methods used to examine subgroups and interactions | 9-10 |
| | | (*c*) Explain how missing data were addressed | N/A |
| | | (*d*) If applicable, explain how loss to follow-up was addressed | N/A |
| | | (*e*) Describe any sensitivity analyses | N/A |
| **Results** | | | |
| Participants | 13* | (a) Report numbers of individuals at each stage of study—eg numbers potentially eligible, examined for eligibility, confirmed eligible, included in the study, completing follow-up, and analysed | 11 |
| | | (b) Give reasons for non-participation at each stage | Figure 1 |
| | | (c) Consider use of a flow diagram | Figure 1 |
| Descriptive data | 14* | (a) Give characteristics of study participants (eg demographic, clinical, social) and information on exposures and potential confounders | 11-12 |
| | | (b) Indicate number of participants with missing data for each variable of interest | |
| | | (c) Summarise follow-up time (eg, average and total amount) | |
| Outcome data | 15* | Report numbers of outcome events or summary measures over time | 11 |

| | | | |
|---|---|---|---|
| Main results | 16 | (*a*) Give unadjusted estimates and, if applicable, confounder-adjusted estimates and their precision (eg, 95% confidence interval). Make clear which confounders were adjusted for and why they were included | 11-14 |
| | | (*b*) Report category boundaries when continuous variables were categorized | 9 |
| | | (*c*) If relevant, consider translating estimates of relative risk into absolute risk for a meaningful time period | N/A |
| Other analyses | 17 | Report other analyses done—eg analyses of subgroups and interactions, and sensitivity analyses | 12 |
| **Discussion** | | | |
| Key results | 18 | Summarise key results with reference to study objectives | 15 |
| Limitations | 19 | Discuss limitations of the study, taking into account sources of potential bias or imprecision. Discuss both direction and magnitude of any potential bias | 17 |
| Interpretation | 20 | Give a cautious overall interpretation of results considering objectives, limitations, multiplicity of analyses, results from similar studies, and other relevant evidence | 15-16 |
| Generalisability | 21 | Discuss the generalisability (external validity) of the study results | 17 |
| **Other information** | | | |
| Funding | 22 | Give the source of funding and the role of the funders for the present study and, if applicable, for the original study on which the present article is based | 18 |
